# The Markov blankets of life: autonomy, active inference and the free energy principle

**DOI:** 10.1098/rsif.2017.0792

**Published:** 2018-01-17

**Authors:** Michael Kirchhoff, Thomas Parr, Ensor Palacios, Karl Friston, Julian Kiverstein

**Affiliations:** 1 Department of Philosophy, University of Wollongong Faculty of Law Humanities and the Arts, Wollongong, New South Wales, Australia; 2 Wellcome Trust Centre for Neuroimaging, London, UK; 3 University of Parma, Parma, Italy; 4 Wellcome Trust Centre for Neuroimaging, Institute of Neurology UCL, London, UK; 5 Department of Psychiatry, AMC, Amsterdam, The Netherlands

**Keywords:** free energy principle, Markov blanket, autonomy, active inference, ensemble Markov blanket

## Abstract

This work addresses the autonomous organization of biological systems. It does so by considering the boundaries of biological systems, from individual cells to *Home sapiens*, in terms of the presence of Markov blankets under the active inference scheme—a corollary of the free energy principle. A Markov blanket defines the boundaries of a system in a statistical sense. Here we consider how a collective of Markov blankets can self-assemble into a global system that itself has a Markov blanket; thereby providing an illustration of how autonomous systems can be understood as having layers of nested and self-sustaining boundaries. This allows us to show that: (i) any living system is a Markov blanketed system and (ii) the boundaries of such systems need not be co-extensive with the biophysical boundaries of a living organism. In other words, autonomous systems are hierarchically composed of Markov blankets of Markov blankets—all the way down to individual cells, all the way up to you and me, and all the way out to include elements of the local environment.

## 1. Introduction

Organisms show a tendency to self-organize into a coherent whole despite them comprising a multiplicity of nested systems. They also continuously work to preserve their individual unity, thus tending to maintain a boundary that separates their internal states from their external milieu ([1]; see also [2]). These tendencies speak to the autonomous organization of biological systems.

This paper addresses the self-organization of autonomous organization in biological systems by asking how Markov blankets of living systems self-organize via active inference—a corollary of the free energy principle. A Markov blanket defines the boundaries of a system (e.g. a cell or a multi-cellular organism) in a statistical sense. It is a statistical partitioning of a system into internal states and external states, where the blanket itself consists of the states that separate the two. The states that constitute the Markov blanket can be further partitioned into active and sensory states. Here, states stand in for any variable that locates the system at a particular point in state space; for example, the position and momentum of all the particles constituting a thermodynamic system—right through to every detail of neuronal activity that might describe the state of the brain. In the thermodynamic example, internal states would correspond to the thermodynamic system (e.g. a gas) in question; the external states would constitute a heat bath; and the Markov blanket could be the states of a container that mediates (directed) exchange between the heat bath and internal states. For an embodied central nervous system, the active and sensory states correspond to the states of all actuators or effectors and sensory organs, respectively.

Statistically, the existence of a Markov blanket means external states are conditionally independent of internal states, and vice versa, as internal and external states can only influence each other via sensory and active states. The presence of the conditional independencies implied by a Markov blanket induces—as shown in Friston [3]—active inference. Active inference, in its simplest formulation, describes the tendency of random dynamical systems to minimize (on average) their free energy, where free energy is an upper bound on (negative) marginal likelihood or evidence (i.e. the probability of finding the system in a particular state, given the system in question). This implies that the kind of self-organization of Markov blankets we consider results in processes that work entirely to optimize evidence, namely self-evidencing dynamics underlying the autonomous organization of life, as we know it. In Bayesian statistics, the evidence is known as ‘model’ evidence, where we can associate the internal states with a model of the external states.

We approach the self-organization of Markov blankets and processes of model optimization that ensue in terms of an optimality principle; namely, the minimization of free energy [4]. Free energy was classically defined in terms of thermodynamic principles, denoting a measure of energy available to a system to do useful work (e.g. maintaining a particular speed in a Watt governor or photosynthesis in plants). The free energy we refer to here is an information-theoretic analogue of the thermodynamic quantity. Free energy is a bound on ‘surprisal’ (or negative model evidence) or more simply ‘surprise’. The time average of surprise is entropy (a measure of uncertainty), so the minimization of free energy through time ensures that entropy is bounded. One can understand surprisal as a measure of how unlikely an observation would be by associating a system's sensory state with an observation or sensory sample [5]. Reducing free energy is therefore the same as optimizing Bayesian model evidence (negative surprisal) for a model (the system) reflected in the probability distributions over sensory data sampled by a system [6]. Crucially, this allows one to explain self-assembly of Markov blankets in terms of approximate Bayesian inference and probabilistic beliefs that are implicit in a system's interactions with its local surroundings [7]. This teleological (Bayesian) interpretation of dynamical behaviour in terms of optimization allows us to think about any system that possesses a Markov blanket as some rudimentary (or possibly sophisticated) ‘agent’ that is optimizing something; namely, the evidence for its own existence. This means we can regard the internal states (and their Markov blanket) as, in some sense, autonomous.

In this paper, we take the internal and active states of a Markov blanket to minimize free energy via active inference. The scope of this formulation is extremely broad. It applies to systems such as coupled pendulums that one would not readily recognize as autonomous. This raises the question of whether the Markov blanket formulation of biological systems is over-broad and thereby explanatorily empty with respect to autonomy. We show that this worry can be handled by formulating a novel distinction between ‘mere active inference’ and ‘adaptive active inference’, as only the latter enables modulation of an organism's sensorimotor coupling to its environment. From adaptive active inference we argue that organisms comprise a multiplicity of Markov blankets, the boundaries of which are neither fixed nor stable. We do this by suggesting that an ensemble of Markov blankets can self-organize into a global or macroscopic system that itself has a Markov blanket. This allows us to provide an illustration of how autonomous systems are realized by multiple self-evidencing and nested Markov blankets. This construction implies that a living system is composed of Markov blankets of Markov blankets [8]—reaching all the way down to cellular organelles and DNA [9] and all the way out to elements of the environment [10].

The paper is organized as follows. In §2 we introduce the Markov blanket concept in the context of active inference under the free energy principle. In §3 we distinguish between mere active inference and adaptive active inference. It is argued that only the latter kind of active inference enables autonomous organization. In §4 we turn to develop the notion of nested Markov blankets, i.e. Markov blankets of Markov blankets.

## 2. The Markov blanket and active inference

**Figure 1.**
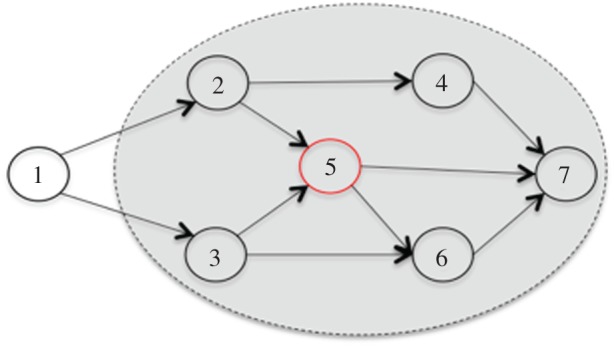
A schematic depiction of a Markov blanket with full conditionals.

A Markov blanket constitutes (in a statistical sense) a boundary that sets something apart from that which it is not. Hence, it is a statistical partitioning of states into internal and external states that are separated by a Markov blanket—comprising active and sensory states. This shows that internal and external states are conditionally independent, as they can only influence one another via active and sensory states. Formally, a Markov blanket renders a set of states, internal and external states, conditionally independent of one another. That is, for any variable *A*, *A* is conditionally independent of *B*, given another variable, *C*, if and only if the probability of *A* and *B* given *C* can be written as *p*(*A|C*) and *p*(*B*|*C*). In other words, *A* is conditionally independent of *B* given *C* if, when *C* is known, knowing *A* provides no further information about *B* [11]. This maps on to the Markov blanket shown in figure 1.

In this figure, the Markov blanket for node {5} is the union of its *parents* {2,3}, the *children* of {5}, which are {6,7}, and the *parents' children* {4}. Hence, {5} = {6,7} U {2,3} U {4} = {2,3,4,6,7}. The union of {5} does not include {1}. This highlights that {1} and {5} are conditionally independent given {2,3,4,6,7}. It also illustrates that, once the union of {5} is given, the probability of {5} will not be affected by the probability of {1}. Formally, {5} is conditionally independent of {1} given {2,3,4,6,7}, if P({5}|{1}, {2,3,4,6,7}) = P({5}|{2,3,4,6,7}). This means that, once all the neighbouring variables for {5} are known, knowing the state of {1} provides no additional information about the state of {5}. It is this kind of statistical neighbourhood for {5} that is called a Markov blanket [12].

The cell is an intuitive example of a living system with a Markov blanket. Without possessing a Markov blanket a cell would no longer be, as there would be no way by which to distinguish it from everything else. This is to say that, if the Markov blanket of a cell deteriorates, there will be no evidence for its existence, and it will cease to exist [13]. This means that the identity of—or the evidence for—any given biological system is conditioned on it having a Markov blanket. So the biological world is a world populated by Markov blankets.

Biological systems have a capacity to maintain low-entropy distributions over their internal states (and their Markov blanket) despite living their lives in changing and uncertain circumstances. This means that biological systems can be cast as engaging in active inference, given that internal and active states of a system with a Markov blanket can be shown to maintain the structural and functional integrity of such a system. To gain some intuition for the motivations behind this formulation, consider that the independencies established by a Markov blanket realize a co-dependence between internal states and external states conditioned on sensory and active states.

The partitioning rule governing Markov blankets illustrates that external states—which are ‘hidden’ behind the Markov blanket—cause sensory states, which influence, but are not themselves influenced by, internal states, while internal states cause active states, which influence, but are not themselves influenced by, external states [7]. Internal and external states can therefore be understood as influencing one another in a continuous and reciprocal fashion, given dependencies between sensory and active states. The independencies established by a Markov blanket are then suggestive of an elemental form of active inference, where internal and active states are directly involved in maintaining the structural and functional integrity of the Markov blanket [7]. This is because active inference rests on the assumption that action—upon which perception depends—minimizes uncertainty or surprise about the causes of an agent's sensory states [14]. Active inference therefore places an upper bound on surprise, i.e. action drives an organism's internal states toward a free energy minima. We develop this point in the remainder of this section.

Active inference is a cornerstone of the free energy principle. This principle states that for organisms to maintain their integrity they must minimize variational free energy. Variational free energy bounds surprise because the former can be shown to be either greater than or equal to the latter. It follows that any organism that minimizes free energy thereby reduces surprise—which is the same as saying that such an organism maximizes evidence for its own model, i.e. its own existence. In other words, self-evidencing behaviour is equivalent to statistical inference [11]. To see this, consider, first,
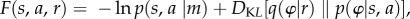
where *s* refers to sensory states, *a* to active states and *r* to internal states. The notation *F*(*s*, *a*, *r*) denotes the variational free energy of internal states and their Markov blanket, 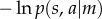 refers to the negative log probability or surprise conditioned on a generative model and 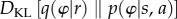 is the Kullback–Leibler (KL) divergence between two probability densities: the variational density, *q*(*φ*|*r*), and the posterior density, *p*(*φ|s*, *a*).

Crucially, this equality gives a Bayesian interpretation of variational free energy. The negative log likelihood or probability is the same as surprise, while the KL divergence measures the discrepancy between the variational density and the true posterior. Minimizing free energy by changing internal states can only reduce the divergence between beliefs about external states (the variational density) and the true posterior density given the states of the Markov blanket. We can think of this as a form of *perception*. Minimizing free energy by changing the active states can only change the surprise or model evidence. This constitutes a form of *action* that underwrites self-evidencing. We now consider this in more detail.

This interpretation means that changing internal states is equivalent to inferring the most probable, hidden causes of sensory signals in terms of expectations about states of the environment. Hidden causes are called hidden because they can only be ‘seen’ indirectly by internal states through the Markov blanket via sensory states. As an example, consider that the most well-known method by which spiders catch prey is via their self-woven, carefully placed and sticky web. Common for web- or niche-constructing spiders is that they are highly vibration sensitive. If we associate vibrations with sensory observations, then it is only in an indirect sense that one can meaningfully say that spiders have ‘access’ to the hidden causes of their sensory world—i.e. to the world of flies and other edible ‘critters’. It is in this sense that one should understand a Markov blanket as establishing a statistical boundary separating internal states from external states. To then act on inferred states of the world means to actively secure evidence that I am what I am; namely, a critter-eating creature.

In a neurobiological setting, Markov blankets can be ‘found’ at each level of the brain's hierarchy, which allows us to associate the brain with a hierarchical Bayesian network—one that is organized such that higher levels in the cortical hierarchy infer (i.e. predict) the states at the level below, all ‘the way down to the changing states of our sensory receptors and physical actuators’ [15, p. 5]. It has proved very helpful to think of exchanges between internal and external states (across the Markov blanket) in terms of a variational free energy-minimizing scheme called predictive coding. In these formulations, free energy can be associated with prediction errors; namely, the difference between sensory states and their prediction is based upon internal states. In predictive coding, predictions are made from the ‘top down’, while prediction error or local surprise is propagated up the hierarchy until any residual error signal is eliminated through updating or parametrizing Bayesian beliefs. This, in turn, enables a system's inferences to acquire a ‘grip’ on the hidden causes of sensory input [16,17].

If we imagine the brain as a hierarchical or nested set of Markov blankets, then the Markov blanket at any particular level in the brain's hierarchy must comprise active and sensory states, where the active states influence lower levels (i.e. external peripheral Markov blankets) and can be regarded as predictions, while prediction errors play the role of sensory states that influence the higher levels (i.e. internal Markov blankets). This coupled exchange of influences minimizes prediction errors at all levels of the hierarchy; thereby constituting (an internalized) form of active inference and implicit free energy minimization.

More generally, belief parametrization is captured by the KL divergence above. This is a measure of the discrepancy between current beliefs (the variational density) and the true posterior distribution. Specifically, it is a measure of the residual (or relative) surprise between the two probability distributions. When the free energy is minimized, the variational density is approximately equal to the posterior distribution. The better this approximation, the smaller the divergence. This means that the variational density approximates exactly the same quantity that Bayesian inference seeks to optimize. This is made clear through the following expression of Bayes' rule:
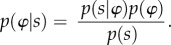


Bayes’ rule states that the (posterior) probability, *p*, of a state, *φ*, given some data (sensations), *s*, is equal to the probability of *s* given *φ* multiplied by the prior probability of *φ*, divided by the prior probability of *s*. The relationship between free energy minimization and Bayes' rule demonstrates that internal states and their Markov blanket can be understood as engaging in approximate Bayesian inference—optimizing their (approximate) posterior beliefs over a model as new sensations are experienced ([18]; for a critique see [15]). This is the Bayesian brain hypothesis [4,19,20].

Active inference reminds us that it is not only internal (e.g. neural) states that perform approximate Bayesian inference, but also active states. This embeds the view of the brain as a Bayesian inference machine within the context of *embodied* (active) inference, formularizing action as the process of selectively sampling sensory data to minimize surprise about their hidden causes ([21]; see also [16,22,23]). To see this, consider that the relative entropy specified by the KL divergence cannot be less than zero. The simplest and most intuitive way by which to illustrate this is that the KL divergence measures how different two distributions are. This means that its minimum should be the point at which the two distributions are equal, i.e. that the difference between the two probability distributions is zero. Mathematically one can show that free energy is an upper bound on surprise by considering the following inequality:
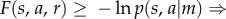

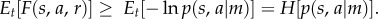


This inequality states that variational free energy bounds surprise, which follows from the fact that the KL divergence cannot be less than zero, i.e. the smallest difference is zero itself. This inequality can also be shown to follow from Jensen's inequality (see appendix A). Moreover, this implies (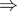): given that the expected (*E*) surprise averaged over time is equal to Shannon entropy, 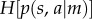, over internal states and their Markov blanket given a generative model, it follows that the expected variational free energy averaged over time, 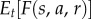, of internal states and their Markov blanket is a bound on entropy. This inequality has several non-trivial implications. We emphasize two below.

First, any system that minimizes entropy by acting to minimize uncertainty about the hidden causes of its sensations must have a model of the kind of regularities it expects to encounter in its environment. This means that, over (phylogenetic and ontogenetic) time, an organism will become a model of its environment (note that natural selection is a form of Bayesian model selection, which will minimize free energy over an evolutionary time scale)—an upshot that is entirely consistent with Conant & Ashby's [24] Good Regulator Theorem. In other words, it suggests that regularities in the environment of an organism become embodied in the organism—if the organism or species persists. Under the free energy principle, this implies that organisms are close to optimal models of their local surroundings, i.e. their niche. Organisms become close to optimal models by minimizing variational free energy, which bounds the evidence for each phenotype or individual model [25]. This does not imply that an agent must (somehow) construct an internal model (i.e. representation) of its outer environment. It simply means that an agent becomes a statistical model of its niche in the sense of coming to embody statistical regularities of its world in its physical and functional composition.

Hence, one should recognize that the morphology, biophysical mechanics and neural architecture of the organism all constitute an agent's model, and that these parameters (or parts) can be tuned and augmented by selection, learning and experience [5]. Consequently, one should not confuse the idea that organisms are models of their niche with the additional view that organisms encode or represent their niche in virtue of being a model. A simple example that illustrates this point is that it is possible to consider the physiological make-up of a fish, say, as a model of the fluid dynamics and other elements that constitute its aquatic environment—its internal dynamics depends on the dynamics of the niche [26]. It is in this embodied sense that one should understand the claim that an organism is a model. In other words, an organism does not merely have a model of its world; rather, it is a model. The model is therefore the entire phenotype [3,21–23,27].

Second, active inference implies that agents are partly responsible for generating the sensory evidence that they garner for themselves. Active inference thus captures the idea that Clark [28], following Lungarella & Sporns [29], calls *information self-structuring.* Information self-structuring highlights the important idea that:

[T]he agent's control architecture (e.g. nervous system) attends to and processes streams of sensory stimulation, and ultimately generates sequences of motor actions which in turn guide the further production and selection of sensory information. [In this way] ‘information structuring’ by motor activity and ‘information processing’ by the neural system are continuously linked to each other through sensorimotor loops. ([29, p. 25]; quoted in [28, p. 18])

We understand this to imply that an agent is able to minimize free energy, and therefore surprise, by actively sampling and changing the hidden causes of its environment. This means that biological systems have expectations and make inferences about the causal regularities and make-up of the environment in which they are situated [30]. In short, given enough time, agents will come to be the authors of the external states (i.e. environments) that reciprocate with predictable, uncertainty resolving sensory feedback of exactly the right sort to sustain cycles of self-evidencing.

## 3. The Markov blanket and adaptive active inference

All Markov blanketed systems can be associated with active inference. In this paper, we wish to not only develop this idea but also analyse what properties a Markov blanketed system must instantiate for it to be autonomous. It is tempting to think that if a system has a Markov blanket—which induces an elemental form of active inference—then that system is by definition an autonomous system. We want to suggest that it be unwise to yield to such a temptation.

### 3.1. The Markov blanket—mere active inference

**Figure 2.**
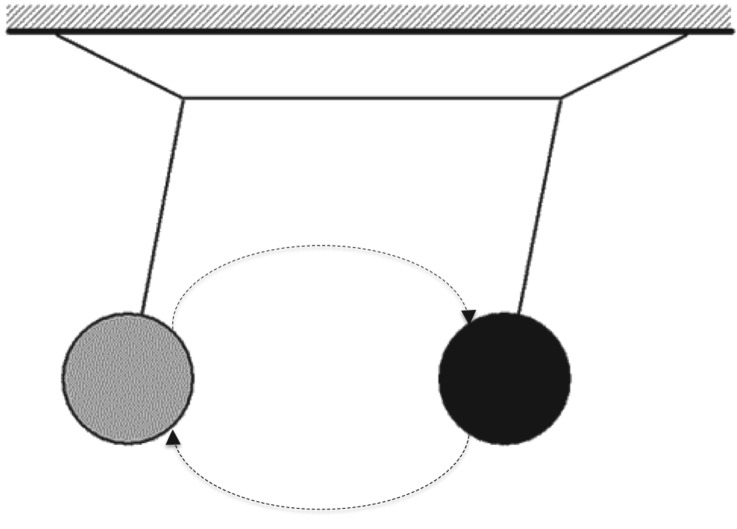
Two oscillating (i.e. coupled random dynamical) systems, A and B, suspended from a beam that is itself able to move. The two arrows illustrate the coupling between pendulum A and pendulum B (for additional discussion, see [16]).

Any Markov blanketed system can be shown to engage in active inference in virtue of its separation of internal and external states (via sensory and active states). Here we consider a very simple example of two coupled random dynamical systems, exemplified by a set of coupled Huygens' pendulums (figure 2).

The beam functions as a Markov blanket. This means that the motions of the two pendulums are statistically independent of one another conditioned on the motion of the beam. If one were to suspend motion of the beam there would be no synchronization between the pendulums. Thus the two pendulums would cease to be dynamically coupled. Furthermore, each pendulum can be understood as a generative model of the other, where the probabilistic mapping from hidden causes (the dynamics of the black clock) to sensory observations (for the grey clock) is mediated by the beam, i.e. the Markov blanket states of the clocks. Note that we are using the terms ‘sensory’ and ‘active’ states in an extremely broad sense, associating active states with position and sensory states with velocity or motion.^1^ This allows us to minimally describe the clocks as engaging in active inference, although of a fairly simple form. We call this *mere active inference*.

What warrants this claim is that it is possible to cast generalized synchrony between two coupled pendulums in terms of mutual information. In information theory, mutual information is the KL divergence between the marginal densities over two sets of variables and the joint distribution. When the two sets of variables are independent, the joint distribution becomes the product of the marginals and the KL divergence or mutual information falls to zero. In virtue of the fact that the states of our pendulums have high mutual information they are effectively obliged to actively infer each other; such that, given the (internal) states of one pendulum, one could infer the (internal) states of the other, which, of course, are the external states of the first. It is in this sense that one can conceive of the two pendulums as engaging in active (Bayesian) inference.

### 3.2. The Markov blanket—adaptive active inference

The dynamics of Huygens' pendulums exemplifies a Markov chain over time. A Markov chain is a special case of a Markov blanket, in which the dependencies among states are restricted to a chain of successive influences with no reciprocal influences or loops. This means that the core properties of a Markov chain do not generalize to all Markov blankets, e.g. the conditional independencies induced by a Markov chain are unidirectional. When applied to successive states over time, Markov chains capture the notion that events are conditionally independent of previous or past events given the current states of the system [12]. Systems with unidirectional conditional independencies are non-autonomous. The reason is that such systems cannot modulate their relation to the world, since a Markov chained system is entirely ‘enslaved’ by its here-and-now—and, in particular, its precedents.

This is not true of biological systems. Biological systems are homeostatic systems that exhibit (or perhaps create) dependencies over multiple time scales. Accordingly, biological systems are able to actively monitor and react to perturbations that challenge homeostatic variables, which may, from time to time, go out of bounds. This means that a biological system must possess a generative model with temporal depth, which, in turn, implies that it can sample among different options and select the option that has the greatest (expected) evidence or least (expected) free energy. The options sampled from are intuitively probabilistic and future oriented. Hence, living systems are able to ‘free’ themselves from their proximal conditions by making inferences about probabilistic future states and acting so as to minimize the expected surprise (i.e. uncertainty) associated with those possible future states. This capacity connects biological qua homeostatic systems with autonomy, as the latter denotes an organism's capacity to regulate its internal milieu in the face of an ever-changing environment. This means that if a system is autonomous it must also be adaptive, where adaptivity refers to an ability to operate differentially in certain circumstances. Were the system not able to do this it would cease to exist [26,31].

The key difference between mere and adaptive active inference rests upon selecting among different actions based upon deep (temporal) generative models that minimize the free energy expected under different courses of action. This is fundamentally different from the generalized synchrony and mere active inference seen in Huygens' pendulums. Imagine that the pendulums could jump around and attach themselves to different beams. In this setting what would happen under adaptive active inference? In fact, the pendulums would aspire to generalized synchrony (i.e. mere active inference) and search out the beams whose tiny movements belied more distal external states (i.e. other pendulums). This reflects the epistemic behaviour that follows from minimizing uncertainty about ‘what's out there’. Clearly, an active pendulum must have a generative model that includes other pendulums suspended from beams. A more heuristic example here would be our tendency to sample salient information that resolves uncertainty about states of the world ‘out there’, e.g. looking for a frown or smile on a person's face. The key point being made here is that there is an autonomy afforded by systems whose active states depend on internal states that parametrize (predictive posterior) beliefs about the consequences of action.

The resulting existential foraging speaks directly to the framework of autopoietic enactivism in naturalist philosophy of mind [22,31–34]. Central to this framework are notions such as *operational closure* and *sense making*.

Operational closure refers to a process of autopoietic self-assembly and self-maintenance separating the internal states of an organism from its external states, providing an organism with an identity. Varela *et al*. [35] highlight this by saying that:

A cell stands out of a molecular soup by creating the boundaries that set it apart from that which it is not. Metabolic processes within the cell determine these boundaries. In this way the cell emerges as a figure out of a chemical background. Should this process of self-production be interrupted, the cellular components … gradually diffuse back into a molecular soup. [35, p. 44]

The very existence of living systems can therefore be construed as a process of boundary conservation, where the boundary of a system is its Markov blanket [8]. This means that the dependencies induced by the presence of a Markov blanket are what keep the system far removed from thermodynamical equilibrium (not to be confused with dynamic equilibrium). In other words, it is the dependencies among states that establish a kinetic barrier, which, in turn, constitutes the system's parts and maintains an energy gradient. The operational closure of any living system speaks directly to the partitioning rule governing Markov blankets; namely that external states may influence internal states even if the former are not constitutive parts of an operationally closed system. Di Paolo [31] makes this explicit, when he says:

[T]here may be processes that are influenced by constituent processes but do not themselves condition any of them and are therefore not part of the operationally-closed network. In their mutual dependence, the network of processes closes upon itself and defines a unity that regenerates itself. [31, pp. 15–16]

Thus, any Markov blanketed system will embody recurrent processes of autopoietic self-generation, which—as long as the system exists—enforces a difference between a living system and everything else [33]. This means that these processes are fundamentally processes of identity constitution, given that they result in a functionally coherent unit [36]. Casting operational closure in terms of the presence of a Markov blanket gives the notion of operational closure a statistical formulation. One of the nice things about casting operational closure in terms of the presence of a Markov blanket is that it allows us to explain what Varela [36] called ‘the intriguing paradox’ of an autonomous identity: how a living system must both distinguish itself from its environment and, at the same time, maintain its energetic coupling to its environment to remain alive. According to Varela: ‘this linkage cannot be detached since it is against this very environment from which the organism arises, comes forth’ [36, p. 78].

The answer to this apparent paradox lies in the conditional independencies induced by the presence of a Markov blanket, which (as we know) separates internal states and external states, and can be further decomposed into active states and internal states. Crucially, active and sensory states are distinguished in the following sense: active states influence but cannot be influenced by external states, while sensory states influence but cannot be influenced by internal states. This constraint enforces conditional independence between internal and external states—from which an autonomous identity can be shown to emerge—while creating a coupling between organism and environment via sensory and active states.

Sense making refers to an organism's possession of operationally closed mechanisms that can ‘potentially distinguish the different virtual (i.e. probabilistic) implications of otherwise equally viable paths of encounters with the environment’ [31, p. 15]. Sense making can therefore be associated with what we call *adaptive active inference*—the idea that living organisms can actively change their relation to their environment. This suggests that living systems can transcend their immediate present state and work towards occupying states with a free energy minimum. This speaks to the main difference between mere active inference and adaptive active inference. Any organism that must adapt to the changing dynamics of its environment must be able to infer the sensorimotor consequences of its own actions. It cannot do so without possessing a generative model of its future states dependent on how its acts. This is what adaptive active inference is: the capacity to infer the results of future actions given a history of previous engagement with the world, harnessed in the prior probabilities reflected in the generative model [37]. Adaptive active inference is therefore inherently associated with hierarchical generative models. Hierarchical generative models comprise nested and multi-layered Markov blankets [38]. The nested structure of such a Markov blanketed system is what induces the multilayered independencies required for a system to realize generative models with temporal and spatial depth, enabling the system to make inference over recursively larger and larger scales of sensorimotor consequences.

Intuitively, to remain alive an organism must avoid crossing terminal species-specific phase boundaries. An example of a phase boundary that makes this clear is the bank of a river. On one side of this boundary, an organism will retain its structural integrity. On the other side, it will not (unless it is amphibious). Being near a riverbank thus presents such an organism with at least two probabilistic outcomes relative to how it might act. It can move in such a way that it falls over the side of the riverbank. Or it can move to remain at some distance to the riverbank. This means that an organism must have prior probabilistic beliefs about (the consequences of) its behaviour, which, in turn, implies that it must be able to sample across different probabilistic outcomes of its own actions. Such an organism instantiates a hierarchically nested generative model consisting of a multiplicity of Markov blankets, the parameters of which are sculpted and maintained during adaptive active inference.

What distinguishes autonomous systems from those lacking autonomy (at least as we have defined autonomy here) is the way that the former makes inferences about action over time [37]. This sheds light on the kind of architectural properties an autonomous system must have for it to successfully restrict itself to a limited number of attracting states. The first observation is that action depends on inference. This means that an organism must be able to make inferences about the outcomes of its own actions. The second observation is that for any organism to make inferences of this kind it must have a generative model of future states. We made this point earlier by stating that an organism must be able to infer the probabilistic outcomes of its own actions. For example, an organism needs to assess what might happen were it to jump into a fast flowing river. Note that such a creature cannot access sensory observations of such outcomes, until it undertakes one action, at the expense of others. This means that systems able to make such future-oriented inferences must possess a generative model with temporal or counterfactual depth [26,39]. A system with a temporally deep generative model will be a system capable of acting (i.e. inferring) ahead of actuality. The deeper the temporal structure of a living system's generative model, the better it will be at sampling across the probabilistic outcomes of its own actions—and the better it will be at entertaining a repertoire of possible actions.

### 3.3. Summary

In summary, active inference is all about maintaining your Markov blanket—a game that can be cast in terms of active inference, under a model of the world that generates sensory impressions or states. This model becomes equipped with prior beliefs that shape action on the world. Generally speaking, active inference assumes that the only self-consistent prior is that the actions undertaken by organisms minimize expected free energy. Or, put differently, organisms will act to minimize expected surprise and thereby resolve uncertainty by actively sampling their environments [14]. There are several intuitive behaviours that emerge under this treatment, which we can illustrate with the riverbank example. Imagine a creature confronted with a riverbank: in the absence of any prior beliefs about what it would be like to be in the water, the river holds an epistemic affordance (i.e. novelty), in the sense that entering the water resolves uncertainty about ‘what would happen if I did that’. If the unfortunate creature subsequently drowned, priors would emerge (with a bit of natural selection) in her conspecifics that water is not a natural habitat. A few generations down the line, the creature, when confronted with a riverbank, will maintain a safe distance in virtue of avoiding expected surprise, i.e. fulfilling the prior belief that ‘creatures like me are not found in water.’

Hence, if a creature cannot swim it becomes imperative to keep away from the banks of the river. This, in turn, implies that its imperative for action selection must be guided by priors stating that whichever action is selected it must be one that minimizes expected surprise. Survival is therefore premised on having a generative model with a particular temporal thickness, underpinning the ergodic property of life (e.g. from now until swimming—or not). Ergodicity implies that the proportion of time an organism is in some state (e.g. on land rather than falling into a river) is the same as the probability of that organism being in that state—assuming that the fewer states the organism visits during its lifetime, the lower its average entropy.

On this view, the ultimate endgame is—perhaps counterintuitively—to become a Huygens' pendulum. In other words, to engineer a world of predictability, harmony and (generalized) synchrony, in which there is no uncertainty about what to do—or what will happen. This aspiration of *adaptive* active inference (namely, *mere* active inference) is famously exemplified by the sea squirt that ‘eats its own brain’ after it has attached itself to the right ‘beam’. One might ask why *Homo sapiens* have failed to reach this existential Nirvana. This is probably due to the fact that the world we populate contains other systems (like ourselves) that confound predictions—in virtue of the deep generative models that lie underneath their Markov blankets. In what follows, we now consider in greater depth the relationships among Markov blankets that endow the world with structure.

## 4. Ensemble Markov blankets: blankets of blankets (of blankets)

In this section, we consider how a collective of Markov blankets can assemble or self-organize into an ensemble that itself has a Markov blanket. Crucially, this allows us to argue for the possibility of two things: namely, that an autonomous system is an operationally closed system with the property of adaptivity, and that this organization is best characterized in terms of Markov blankets of Markov blankets, i.e. ensemble Markov blankets. Active inference can therefore make sense of complex living systems whose autonomy can be described at multiple levels of organization.^2^

One of the key characteristics of all living systems is their hierarchical nature. This means that a non-trivial property of life is its propensity to form multi-level and multi-scale structures of structures [30]. Crucially, each of these systems makes up a larger whole with respect to its parts, while, at the same time, being a part of an even larger whole, and so on. Cells assemble to form tissues, tissues combine to form organs, and organs organize into organisms. These nested, multi-layered systems are, in turn, embedded within even larger social systems and ecosystems. Indeed, over the entire living world, we find living systems organized into even larger living systems [1]. This view of systems embedded within systems lies at the heart of systems thinking in biology [40,41] and neuroscience [42,43], and has its developmental roots in synergetics [44] and thermodynamics [45].

**Figure 3.**
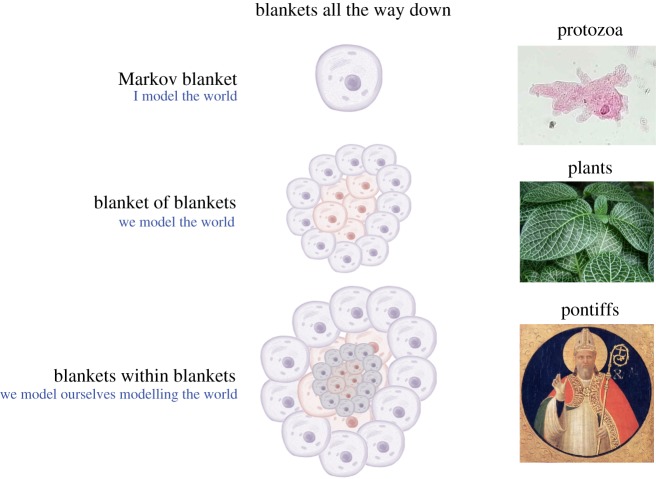
Nested Markov blankets of Markov blankets at different levels of organization.

Any one of these systems has its unique Markov blanket. This means that life comprises Markov blankets of Markov blankets—all the way down to cellular organelles and molecules like DNA, and all the way up to organisms and their environments, both ecological and social (figure 3).

A compelling reason for making this claim is that it allows us to describe systems at multiple different levels. That individuals can be distinguished from one another implies that each system has a Markov blanket—e.g. that the organs within an organism can be distinguished implies that they exist. Note that the upshot of this line of thinking is that an autonomous system (any system able to remain far removed from its terminal phase boundaries) has a Markov blanket at its superordinate level composed of Markov blankets at its supraordinate level. The self-evidencing dynamics of autonomous organization can therefore be cast as exhibiting two different yet complementary tendencies: an *integrative tendency* of a multiplicity of Markov blankets to self-organize into a coherent self-evidencing whole, and a *self-assertive tendency* to preserve individual autonomy. This is the basis for the claim that autonomous systems are made up of Markov blankets of Markov blankets.

Central to the idea of an ensemble Markov blanket is that the statistical form and subsequent partitioning rule governing Markov blankets allow for the formation of Markov blankets at larger and larger scales (of cells, of organs, of individuals, of local environments). The reason for this is that the organization of Markov blankets occurs recursively at larger and larger scales, recapitulating the statistical form of Markov blankets at smaller microscopic scales (figure 3).

**Figure 4.**
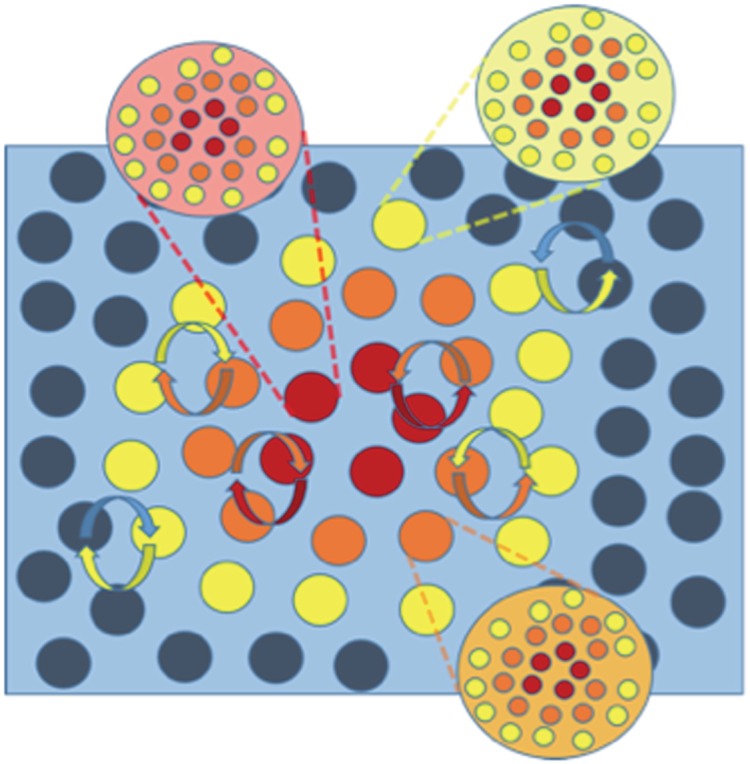
Markov blankets of Markov blankets. This illustrates how the conditional dependency structure of Markov blankets can be replicated at larger spatial scales. Internal (red) states are separated from external (blue) states via sensory (yellow) states and active (orange) states at different scales of organization [46].

Figure 4 depicts a system constituted by a multiplicity of nested Markov blankets at the scale of microscopic dynamics, and a larger or bigger Markov blanket at the macroscopic scale of collective dynamics. It thus becomes possible to distinguish between internal and external states only by appeal to the presence of a third set of states; namely, the Markov blanket. This means that the assembly of Markov blankets can be understood to occur in a nested and hierarchical fashion, where a Markov blanket and its internal states at the macroscopic scale consist of smaller Markov blankets and their internal states at microscopic scales of systemic operations. Crucially, the conservation of Markov blankets (of Markov blankets) at every hierarchical scale enables the dynamics of the states at one scale to enslave the (states of) Markov blankets at the scale below, thereby ensuring that the organization as a whole is involved in the minimization of variational free energy. It is thus only when the properties of the collective dynamics feed back into the scale below, forming a free energy-minimizing system at the scale of the whole system, that it is possible to talk meaningfully of ensemble Markov blankets—blankets whose self-evidencing dynamics result in an overall self-sustaining organization.

We can explain the nested Markov blanket organization of living systems further by appeal to basic principles of complexity theory. The first is that the existence of a superordinate Markov blanket organization, which is intimately connected to the idea of an *order parameter* in complexity theory [47]. An order parameter is a macroscopic (global or systemic) feature of a system, and captures the coherency (i.e. dependencies) among the parts making it up. In this context, the statistical form of each constituent means that each part infers that it is an internal state of a larger Markov blanket, which, in turn, allows each internal state to influence and be influenced by all other internal states. This process of self-assembling Markov blankets of Markov blankets must thus be understood as reconfiguring the particular dependencies between internal, external, active and sensory states. The second is that this kind of self-assembling activity implies a separation of the dynamics involved into *slow and fast time scales*—a signature feature of the slaving principle in synergetics [48]. As a result it becomes possible to understand that slow ensemble dynamics arise from microscale dynamics unfolding over fast time scales. But notice that since the ensemble Markov blanket plays the role of an order parameter it follows that all the dynamics at the microscale no longer behave independently but ‘are sucked into an ordered coordinated pattern’ [49, p. 8]. The dynamics at the microscale are therefore constrained by the dynamics at the macroscale. A famous example of this is the Belousov–Zhabotinsky reaction in chemistry; however, there are many other examples in the literature on complex systems and self-organization (see, for example, [47,49–54]). Any autonomous agent will therefore be made up of many Markov blankets, the dynamics of which unfolds on different temporal and spatial scales.

In some cases, it will be correct to identify the boundaries of an autonomous organization with the biophysical boundaries of a single individual. The cell is an obvious case. Its intracellular web of networks is separated from its extracellular environment by a Markov blanket. However, the organization of Markov blankets of Markov blankets can also extend in an outward direction. In such circumstances, it is more appropriate to conceive of the realizers of Markov blanketed systems as including extra-individual features of an organism's local environment.

The water boatman is an example of an autonomous system, the internal states of which comprise environmental aspects [31]. The water boatman is ‘able to breathe underwater by trapping air bubbles (plastrons) using tiny hairs in the abdomen. The bubbles even refill with oxygen due to the differences in partial pressure provoked by respiration and potentially can work indefinitely. They provide access to longer periods underwater thanks to a mediated regulation of environmental coupling (which is nevertheless potentially riskier than normal breathing)’ [31, p. 17]. This example highlights the fact that some creatures *incorporate* elements of their niche that jointly contribute to—or preserve—their structural integrity over time. Hence, the bubbles in which the boatman is wrapped are best conceived of as elements constituting the boatman's Markov blanket. This follows because without the bubbles the water boatman would not be able to minimize variational free energy. It is in this sense that the water boatman *plus* its self-orchestrated air bubbles constitute a Markov blanket the boundaries of which reach all the way out to include parts of the environment.

Markov blankets of autonomous systems are not merely capable of extending outwards. The boundaries can also be shown to be malleable. This is the case with Clark's [9] example of the caterpillar-cum-butterfly. Most caterpillars will spend part of their lives on their food source, devouring it. Over the course of their lifespan, caterpillars move away from their preferred source of food. They do this to find shelter—a place in which to pupate, the process that transforms them into adulthood. In all caterpillars pupation occurs inside a protective shell known as a chrysalis, which is assembled by the caterpillar literally shedding its skin. It is this self-made shell that protects the caterpillar while it morphs into a butterfly. Fascinatingly, during this phase transition most of the caterpillar's body breaks down to a mass of undifferentiated cells—like a primordial soup out of which cells begin to set themselves apart and self-organize into a new phenotypic form. When the transformation is complete—a process known as holometabolism—the caterpillar turned pupa emerges in the form of a butterfly. From a certain point of view, these phase transitions may look as if the organism is unable to maximize evidence for its own autonomy—for its own existence. Yet, as Clark (convincingly, in our view) argues, ‘the act of transformation is itself an essential part of the on-going project of exchanging entropy with the environment so as to persist in the face of the second law [of thermodynamics]’ [9, p. 12]. This means that the succession of differently Markov blanketed organizations is itself a free energy-minimizing strategy—one that occurs over the entire life cycle from caterpillar to butterfly. As Clark puts it: ‘The life-cycle is self-evidencing insofar as the very existence of the linked stages (caterpillar, pupa, butterfly) provides evidence for the “model” that is the metamorphic agent, where that agent is not identified with a specific morphology (which would correspond merely to one state of the life cycle) but with the temporally extended whole’ [9, p. 12].

These examples both show that the organizational boundaries of living systems are open and flexible in the precise sense that such boundaries need not be co-extensive with an organism's bodily boundaries.

## 5. Conclusion

In this paper we have argued that the autonomous organization of living systems consists of the hierarchical assembly of Markov blankets of Markov blankets through adaptive active inference. We have further argued that this nested Markov blanketed organization need not be co-extensive with the biophysical boundaries of the organism but may extend to include aspects of an organism's environment. We have not established (i.e. shown) that the self-organization of hierarchically composed Markov blankets of Markov blankets is an emergent property of systems (under the right sorts of conditions). Our focus in this paper has been on the implications of such an emergent structure. Having said this, in a parallel (formal) programme of work, we have used simulations to provide a proof of principle that this particular (and possible ubiquitous) form of self-organization is an emergent property. The simulations we use in this parallel work build on previous work that characterizes the emergence of pattern formation and morphogenesis in a biological system [7] but with an important twist. Instead of simulating the assembly of Markov blankets systems from the bottom up, we apply a top-down approach (see Palacios *et al.* [46] for details).

## Figures and Tables

**Figure 5.**
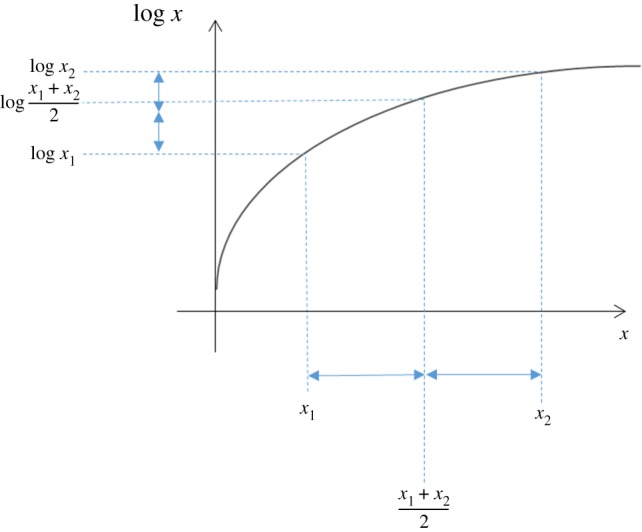
Illustration of Jensen's inequality used to show that the free energy is an upper bound on surprise.

## Endnotes

1 This speaks to a subtle argument that, briefly, goes as follows. The position states of one Markov blanket can only influence the velocity states of another (through forces). This ensures that position (i.e. action) states of one blanket cannot be directly influenced by velocity (i.e. sensory) states of another blanket (i.e. external states).

2 A slightly more technical motivation for Markov blankets of Markov blankets follows from the notion of states. We predicated our argument on the ensemble dynamics of states to show that things are only defined in terms of their Markov blankets. It therefore follows that the states of things are states of Markov blankets. From this it follows that Markov blankets of states must entail Markov blankets of (the states of) Markov blankets.

## Data accessibility

This article has no additional data.

## Appendix A

The curve of a logarithmic function is concave. This has an important implication when averages are taken either of the logarithmic function or of the input to the function. To gain some intuition for this, consider a dataset of just two points, *x*_1_ and *x*_2_. The average of these will lie halfway between the two, as shown on the *x*-axis in figure 5. When the logarithms of *x*_1_, *x*_2_ and their average are calculated (*y*-axis), it is clear that the log of the average lies above the midpoint between log*x*_1_ and log*x*_2_. The midpoint is the average of the logarithms, so this implies that *the log of an average is greater than the average of a log*. This is a statement of Jensen's inequality.

If we replace the variable *x* with a ratio of probabilities, , and take averages (‘expectations’) with respect to a distribution *Q*(*s*), we can use Jensen's inequality to write:

The term on the left (the average of the log) is the negative free energy. The term on the right is the negative surprise. To see this, if we write the expectation out in full, we have

These allow us to rewrite the inequality (noting that, when we make both sides negative, the inequality sign reverses),

This is the statement that the free energy is an upper bound on surprise.
